# Evidence of the Dysbiotic Effect of Psychotropics on Gut Microbiota and Capacity of Probiotics to Alleviate Related Dysbiosis in a Model of the Human Colon

**DOI:** 10.3390/ijms24087326

**Published:** 2023-04-15

**Authors:** Yasmina Ait Chait, Walid Mottawea, Thomas A. Tompkins, Riadh Hammami

**Affiliations:** 1 NuGut Research Platform, School of Nutrition Sciences, Faculty of Health Sciences, University of Ottawa, Ottawa, ON K1N 6N5, Canadawmott020@uottawa.ca (W.M.); 2 Department of Microbiology and Immunology, Faculty of Pharmacy, Mansoura University, Mansoura 35516, Egypt; 3 Lallemand Bio-Ingredients, Montreal, QC H1W 2N8, Canada; 4 Department of Biochemistry, Microbiology and Immunology, Faculty of Medicine, University of Ottawa, Ottawa, ON K1N 6N5, Canada

**Keywords:** gut microbiome, psychotropics, aripiprazole, (S)-citalopram, gut dysbiosis, probiotics

## Abstract

Growing evidence indicates that non-antibiotic therapeutics significantly impact human health by modulating gut microbiome composition and metabolism. In this study, we investigated the impact of two psychotropic drugs, aripiprazole and (S)-citalopram, on gut microbiome composition and its metabolic activity, as well as the potential of probiotics to attenuate related dysbiosis using an ex vivo model of the human colon. After 48 h of fermentation, the two psychotropics demonstrated distinct modulatory effects on the gut microbiome. Aripiprazole, at the phylum level, significantly decreased the relative abundances of Firmicutes and Actinobacteria, while increasing the proportion of Proteobacteria. Moreover, the families *Lachnospiraceae*, *Lactobacillaceae,* and *Erysipelotrichaceae* were also reduced by aripiprazole treatment compared to the control group. In addition, aripiprazole lowered the levels of butyrate, propionate, and acetate, as measured by gas chromatography (GC). On the other hand, (S)-citalopram increased the alpha diversity of microbial taxa, with no differences observed between groups at the family and genus level. Furthermore, a probiotic combination of *Lacticaseibacillus rhamnosus* HA-114 and *Bifidobacterium longum* R0175 alleviated gut microbiome alterations and increased the production of short-chain fatty acids to a similar level as the control. These findings provide compelling evidence that psychotropics modulate the composition and function of the gut microbiome, while the probiotic can mitigate related dysbiosis.

## 1. Introduction

Considerable evidence suggests that the gut microbiome interacts with the brain and plays a key role in the pathogenesis of mental illnesses [1,2]. Multiple studies investigating disease-specific microbiome species biomarkers have reported, albeit confusing, changes in microbiome abundance associated with mental diseases [3,4]. For instance, Mason et al. [5] recently reported the depletion of *C. leptum* and *Bacteroides* in depression and anxiety. A recent meta-analysis of 34 studies reported that the gut microbiota is characterized by a transdiagnostic perturbation pattern among different psychiatric disorders, including depression, bipolar disorder, schizophrenia, and anxiety [4]. This pattern is characterized by the depletion of anti-inflammatory butyrate-producing bacteria, such as *Faecalibacterium* and *Coprococcus*, along with the enrichment of pro-inflammatory members such as *Eggerthella* [4]. However, no information has been reported regarding medications as confounding factors.

A growing body of research has recently demonstrated that several pharmaceutical non-antibiotic compounds, including psychotropics, drugs used to treat mental illnesses, influence human gut microbiota and/or isolated microbial strains [6,7]. Indeed, psychotropics have been reported to exhibit a high potential antimicrobial effect against individual isolates of gut microbiota, similar to antibiotics, through which they can alter the microbiome composition [6,7,8]. Therefore, gut microbiome alterations in mental disorders may be directly related to disease pathogenesis or chronic use of psychotropic medications, which could be undervalued confounding factors [6]. For example, *Faecalibacterium prausnitzii*, *Akkermansia muciniphila*, *Clostridium leptum*, and *Bacteroides fragilis*, important anti-inflammatory microbial groups linked to many disorders, were found to be significantly affected by several classes of psychotropics [6]. Therefore, the antimicrobial effect of psychotropic drugs should be considered a factor of utmost importance, knowing that patients with mental disorders are generally on long-term medication.

While there is evidence of the antimicrobial effects of some psychotropics on isolated strains or animal models’ gut microbiota, a comprehensive understanding of the response of the human gut microbiota to psychotropics is still lacking. To address this, an ex vivo fermentation model was employed in this study to investigate the impact of psychotropic drugs on the structure and functionality of the human gut microbiome without host effects. Ex vivo models of the colon microbiome, from simple short-term batch fermentation to multistage long-term continuous flow models, provide a highly controlled environment (retention time, pH, temperature, anaerobiosis, and medium composition) to study the effects of xenobiotic compounds on gut microbiota [9]. In particular, continuous fermentation systems with immobilized gut microbiota have been shown to simulate the high cell density, biodiversity, and long-term stability of the intestinal microbiota [10,11].

Numerous strategies have been proposed to restore the balance of the dysbiotic microbiome due to psychotropic medications [6,12]. Many previous studies have supported the role of probiotics (single- or multi-strain) in preventing and treating antibiotic-associated diarrhea and antibiotic-related dysbiosis by restoring the balance of the intestinal microbiome [13,14]. For example, *Lacticaseibacillus rhamnosus* NK210 and *Bifidobacterium longum* NK219 were found to alleviate gut dysbiosis, immune imbalance, and cognitive impairment associated with antibiotic or lipopolysaccharide (LPS) use [15]. Therefore, interventions with probiotics have been suggested as a potential strategy to improve microbiome composition and reduce the adverse effects of psychotropics [12]. Our previous study [6] has illustrated the antimicrobial activities of psychotropics against common members of the gut microbiota, with aripiprazole and (S)-citalopram identified as potent antimicrobial psychotropics. Therefore, this study aimed to investigate the impact of two potent antimicrobial psychotropic drugs, aripiprazole and (S)-citalopram, on human colonic microbiome diversity and metabolism, using an ex vivo model of the human colon. In addition, the growth and capacity of a probiotic mixture consisting of *Lacticaseibacillus rhamnosus* HA-114 and *Bifidobacterium longum* R0175 to alleviate psychotropics-related dysbiosis were assessed.

## 2. Results

### 2.1. Psychotropics Alter the Diversity of the Gut Microbiome

**Figure 1:**
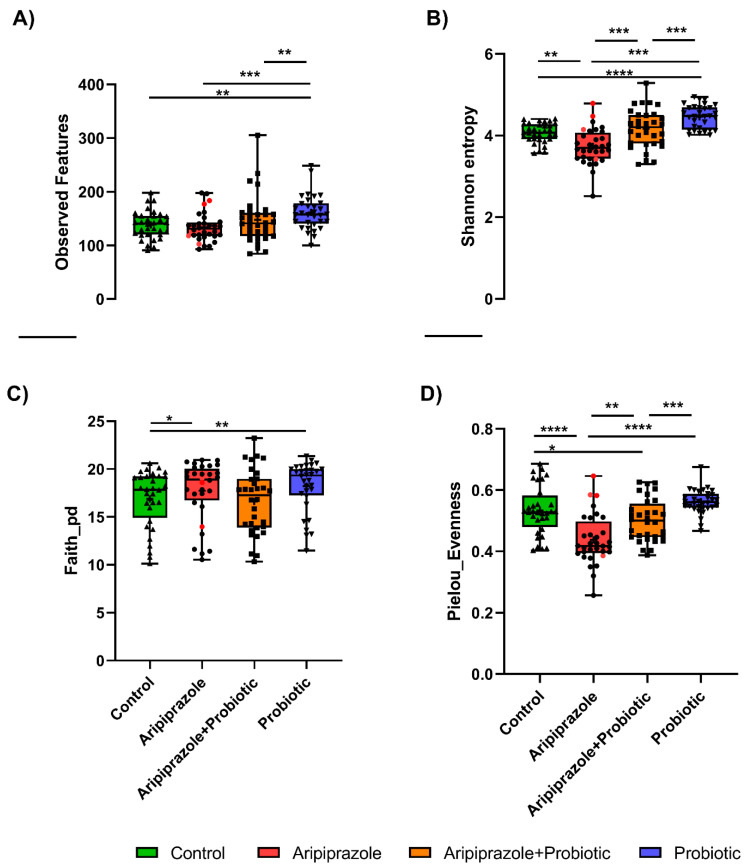
Modulation of microbiota composition following treatment with aripiprazole and probiotics. (**A**) observed features, (**B**) Shannon index, (**C**) Faith’s phylogenetic diversity, and (**D**) Pielou evenness of all treated samples (*n* = four biological replicates each). Results were calculated from rarefied 12,000 reads per sample using QIIME2. 2020.6 version. Middle lines represent the mean. Data were analyzed using the Kruskal- Wallis test and Two-stage Benjamini, Krieger, and Yekutieli FDR procedure; * *p* < 0.05, ** *p* < 0.01, *** *p* < 0.001, **** *p* < 0.0001. Red dots represent the zero time points.

**Figure 2:**
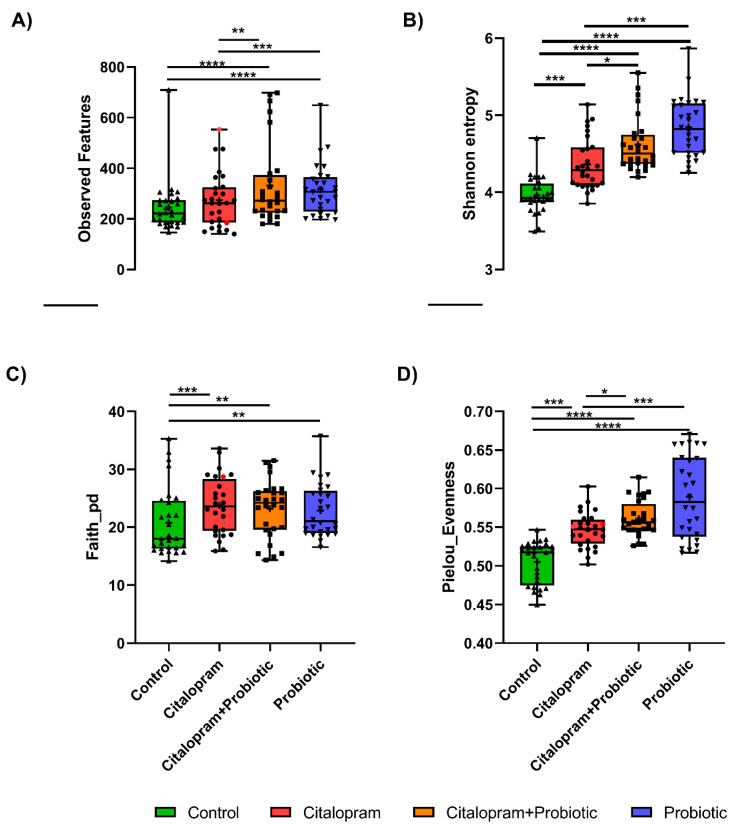
Modulation of microbiota composition following treatment with (S)-citalopram and probiotics. (**A**) observed features, (**B**) Shannon index, (**C**) Faith’s phylogenetic diversity, and (**D**) Pielou evenness of all treated samples (*n* = four biological replicates each). Results were calculated from rarefied 12,000 reads per sample using QIIME2. 2020.6 version. Middle lines represent the mean. Data were analyzed using the Kruskal- Wallis test and Two-stage Benjamini, Krieger, and Yekutieli FDR procedure; * *p* < 0.05, ** *p* < 0.01, *** *p* < 0.001, **** *p* < 0.0001. Red dots represent the zero time points.

**Figure 3:**
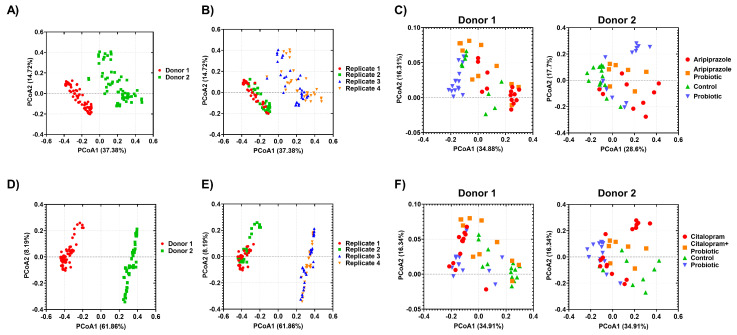
Plots of Principal Coordinate Analysis (PCoA) based on Bray-Curtis distances among the identified microbiota in different samples. (**A**–**C**): effect of aripiprazole treatment; (**D**–**F**) effect of (S)-citalopram treatment. The PCoA figures show clustering based on source donor (**A**,**D**); experiment replicate (**B**,**E**) and treatments of each donor microbiota (**C**,**F**). The samples were colored as indicated in legends. PCoA1 and PCoA2 represent the top two coordinates that captured the highest microbial variability among samples, and the percentage shown indicates the fraction of variation represented by each coordinate.

The alpha diversity (observed features, Shannon entropy, Faith-pd, and Peilou-Evenness) were evaluated for the microbiota from the four bioreactors between 0 and 48 h after treatment with aripiprazole or (S)-citalopram and probiotics (Figure 1 and Figure 2). The diversity of the total bacterial community decreased significantly during the 48 h of treatment with aripiprazole as indicated by observed features and Shannon entropy indices (Figure 1A,B). Aripiprazole treatment significantly reduced bacteria species’ evenness in all statistical comparisons (Figure 1D). However, bacterial species richness, indicated by Faith-pd indices, was not affected by aripiprazole administration (Figure 1C). The probiotics addition also increased the diversity of gut microbiota compared to the group treated with aripiprazole alone or in combination with probiotics (Figure 1). On the other hand, the treatment with (S)-citalopram did not show a consistent effect on the alpha diversity of the microbial community displayed by its evenness and richness (Figure 2A–D). The Shannon entropy and Peilou-Evenness indices increased significantly in the groups treated by (S)-citalopram and (S)-citalopram + probiotics as compared to the control group (Figure 2B,D). Concurrently, the Faith-pd indices value in the (S)-citalopram and (S)-citalopram + probiotics treatment groups were significantly higher than the control group (Figure 2C). Again, the addition of a probiotics mixture increased the alpha diversity of the gut microbiota compared to the (S)-citalopram alone or (S)-citalopram + probiotics treatment groups (Figure 2). Additionally, Beta diversity was assessed based on Bray-Curtis distances and presented through principal coordinates analysis (PCoA) (Figure 3). Results revealed that the microbiota communities from the two donors were highly distinct, forming two separate clusters (*p* = 0.001; Figure 3A,D). In addition, (PCoA) revealed a clear separation between experimental replicates within each donor (*p* = 0.001; Figure 3B,E) and distinct clustering of the aripiprazole or (S)-citalopram and other treatment groups (*p* = 0.001; Figure 3C,F). Statistics of the employed ADONIS multivariate analysis of variance of beta diversity significance are summarized in Supplementary Table S1.

### 2.2. Effect of Psychotropics Alone or in Combination with Probiotics on Gut Microbiome Composition

**Figure 4:**
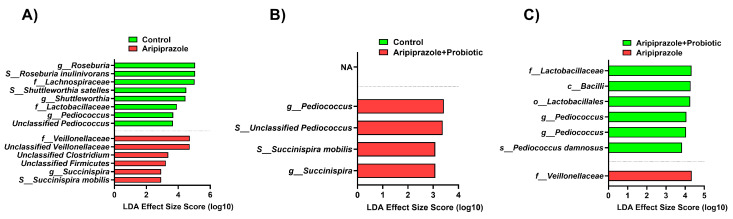
Histograms of the linear discriminant analysis (LDA) scores showing microbial taxa that vary significantly in abundance between: (**A**) no-treatment control and aripiprazole treatment, (**B**) no-treatment control and aripiprazole + probiotic treatment, and (**C**) aripiprazole and aripiprazole + probiotic treatments.

**Figure 5:**
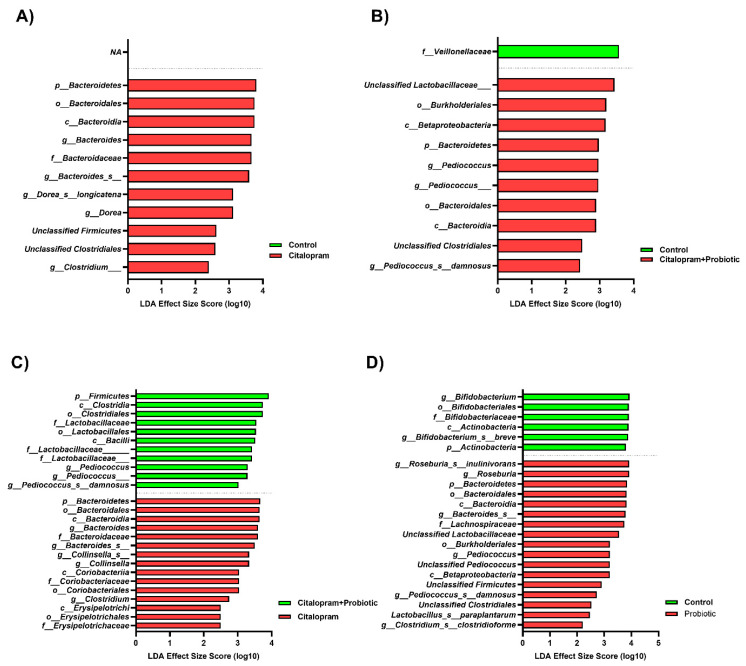
Histograms of the linear discriminant analysis (LDA) scores showing microbial taxa that vary significantly in abundance between: (**A**) no-treatment control and (S)-citalopram treatment, (**B**) no-treatment control and (S)-citalopram + probiotic treatment, (**C**) (S)-citalopram and (S)-citalopram + probiotic treatments, (**D**) no-treatment control and probiotic treatment.

The composition of gut microbiota was evaluated according to treatment. The relative abundance kinetics of the intestinal microbiota in the different treatment groups are presented in Supplementary Figure S1A,B. The most identified phyla among the different treatment groups comprised *Firmicutes*, *Actinobacteria*, *Proteobacteria*, and *Bacteroidetes*, with *Firmicutes* and *Actinobacteria* being the dominant phyla at different time points. The aripiprazole treatment significantly decreased the relative abundance of Firmicutes and Actinobacteria (*p* < 0.05), while significantly increased *Proteobacteria* abundance starting at 6 h to 24 h of treatment, compared to the control (Figure S1A). The simultaneous addition of a combined probiotic mixture and aripiprazole to bioreactors significantly attenuated the drug’s inhibitory effect by increasing the level of *Firmicutes* and *Actinobacteria* while decreasing the abundance of *Proteobacteria* phylum, compared to Aripiprazole treatment alone. Compared to aripiprazole, the relative abundance of Bacteroidetes was increased in the bioreactor treated with (S)-citalopram, but no significant effect in the other phyla was observed following the same treatment (Figure S1B). In keeping with the observations mentioned above, the Linear discriminate analysis (LDA) effect size calculation showed that the aripiprazole treatment led to a major shift in microbiota composition, as illustrated in Figure 4. At the family level, *Lachnospiraceae*, and *Lactobacillaceae* were three families that showed a decreasing trend following aripiprazole treatment (Figure 4A). At the genus level, *Roseburia* and *Pediococcus* genera were significantly depleted after administering aripiprazole compared to control (Figure 4A). The combined addition of aripiprazole and probiotics increased the abundance of *Pediococcus* and *Succinspira* as compared to the control (Figure 4B). The microbiota supplemented with the probiotic mixture showed enrichment in *Lactobacillaceae* and *Pediococcus* compared to aripiprazole treatments (Figure 4C). On the other hand, (S)-citalopram addition induced significant changes in *Bacteroides*, *Dorea*, and *Clostridium* compared to the control (Figure 5A). Combined treatment of (S)-citalopram with the probiotic mainly increased the abundance of *Pediococcus* and Bacteroidetes spp (Figure 5B). Moreover, an apparent enrichment was observed in the Firmicutes phylum, *Lactobacillaceae,* and *Bifidobacteriaceae,* families as well as in the *Pediococcus* and *Bifidobacterium* at the genus level, when the microbiota is supplemented with the probiotic mixture as compared to baseline control and (S)-citalopram treatments (Figure 5C,D).

### 2.3. Probiotic Strains Resist the Psychotropics’ Antimicrobial Effect as Illustrated by qPCR Analysis

As *L. rhamnosus* HA-114 and *B. longum* R0175 were included as a probiotic mixture in the treatment, qPCR analysis coupled with propidium monoazide (PMA) treatment was used to detect viable bacteria of these two strains and quantify their absolute abundance. As expected, the strains were detected only in microbiota supplemented with the probiotic mixture. Their abundance remained stable (≈8 log CFU/mL) and did not significantly differ from the theoretical washout curve over the 48 h of treatment (Supplementary Tables S2 and S4). We can clearly observe that the addition of the probiotic mixture with the tested psychotropics did not affect their growth in the bioreactors within the treatment period (Supplementary Tables S3 and S5).

### 2.4. Effect of Psychotropics Alone or in Combination with Probiotics on Microbiota SCFAs Metabolism

**Figure 6:**
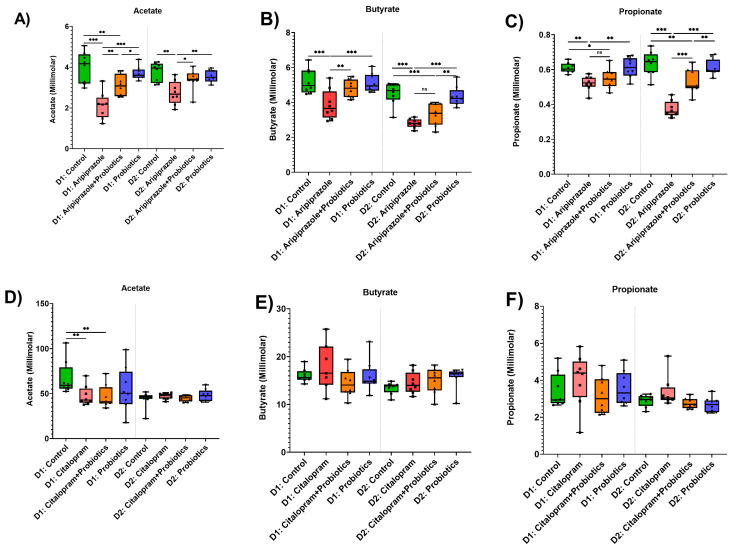
Short-chain fatty acids (SCFAs) concentration was measured by GC over 48 h with all treatment groups with aripiprazole (**A**–**C**) or Citalopram (**D**–**F**). (**A**,**D**) acetate, (**B**,**E**) butyrate, and (**C**,**F**) propionate. Each time point is represented with 4 biological replicates × 2 technical measures. Different treatments are compared statistically for each donor using one way ANOVA test followed by correction for multiple comparisons by controlling false discovery rate using the two-stage step up procedure of Benjamini Krieger and Yekutieli. * *p* < 0.05, ** *p* < 0.01, *** *p* < 0.001.

To further understand the effect of aripiprazole, we used the GC method to determine microbiota SCFAs changes. The three major SCFAs generated by gut microbiota, namely butyrate, acetate, and propionate, were identified and quantified in all treatment groups with aripiprazole or (S)-citalopram. As shown in Figure 6A–C), all identified SCFAs concentrations were significantly decreased (*p* < 0.05) during exposure to aripiprazole compared to the control group. When the probiotic mixture was added to the microbiota, the concentrations of SCFAs were higher than the aripiprazole group and similar to the control group (Figure 6A–C; *p* < 0.05). The (S)-citalopram treatment decreased the acetate concentrations over 48 h of incubation, while the butyrate and propionate concentrations remained constant without any significant changes compared to the control (Figure 6D–F). Likewise, the probiotic addition tends to bring the concentrations of SCFAs to the control level compared to (S)-citalopram treatment.

## 3. Discussion

This study aimed to investigate the effects of aripiprazole and (S)-citalopram, two commonly prescribed psychotropic drugs, on the human gut microbiome using an ex vivo continuous fermentation system that mimicked the conditions in the adult proximal colon. This study confirmed previous findings [6] that psychotropic medications could induce significant changes in the gut microbiota, leading to dysbiosis due to their potent antimicrobial properties. Aripiprazole, an atypical antipsychotic primarily used to treat schizophrenia and control agitation and disturbed behavior [16], was found to shift the microbiome composition and reduce its alpha diversity, as determined by 16S rDNA sequencing. These findings are consistent with previous in vitro and in vivo studies that demonstrated the inhibitory effects of aripiprazole and other psychotropics on gut microbiome growth and their ability to modulate its structure [7,8,17].

At the phylum level, treatment with aripiprazole has been found to cause an increased abundance of *Proteobacteria* and decreased the prevalence of *Firmicutes* and *Actinobacteria*. *Proteobacteria* are typically found in low abundance in healthy humans, and an increase in their abundance has been suggested as a potential diagnostic criterion for gut dysbiosis and disease [18]. Furthermore, an increase in *Proteobacteria* can lead to the gut microbiota entering a pathogenic state, making it more susceptible to infections by exogenous pathogenic microbes [19]. *Firmicutes*, on the other hand, including families such as *Lachnospiraceae* and *Lactobacillaceae* and genera such as *Roseburia*, were most suppressed by aripiprazole treatment. *Lachnospiraceae* are part of the core gut microbiome and are among the main SCFAs producers, with *Roseburia* species often associated with a healthy state [20]. Additionally, *Roseburia* and *Blautia* species are among the genera most involved in regulating gut inflammation, atherosclerosis, and maturation of the immune system through butyrate metabolism [21]. Multiple members of the *Lachnospiraceae* family have also been reported to be significantly inversely correlated with major depressive disorder (MDD) [22]. While the relative abundance of *Erysipelotrichaceae* was shown to be significantly lower in patients with MDD than in a healthy population [23], other reports found a positive correlation between the relative abundance of *Erysipelotrichaceae* and Parkinson’s disease [24]. The antidepressant (S)-citalopram, a selective serotonin reuptake inhibitor (SSRI), modulated the gut microbiota differently than aripiprazole. While aripiprazole led to a decrease in alpha diversity and a significant shift in microbial taxa at the phylum and family levels, (S)-citalopram tended to increase alpha diversity without differences in taxon abundance at the family and genus levels. These results are consistent with a recent study that investigated the effects of escitalopram ((S)-citalopram enantiomer) (therapeutic dose of 5–20 mg) on the gut microbiota of hospitalized patients with depressive episodes, revealing a significant increase in fecal microbiota alpha diversity without changes in taxa abundance after six weeks of treatment [25].

The simultaneous addition of the probiotic mixture of *L. rhamnosus* HA-114 and *B. longum* R0175 with aripiprazole/(S)-citalopram had a protective effect on the gut microbiota, helping maintain diversity levels close to those of the control group. Previous studies have reported that probiotics can alleviate gut microbiota impairment due to antibiotic treatment and help the host restore homeostasis after perturbation [26,27,28]. Other studies have shown promising positive effects of probiotics on the gut microbiota [29]. Given the similarities between several psychotropics and antibiotics in terms of chemical structure, mechanisms of action, etc., probiotics may have a positive role in alleviating drug-induced microbiota dysbiosis [12]. Our study provides the first evidence of the potential of probiotics in alleviating psychotropic-related microbiome dysbiosis.

Microbial SCFAs have been shown to contribute significantly to host health within the gut and periphery [30]. In our study, we found that the administration of aripiprazole and (S)-citalopram led to a decrease in the concentrations of three main SCFAs (butyrate, acetate, and propionate), most likely due to the depletion of SCFA-producing bacteria. However, supplementation with the probiotic mixture significantly increased the levels of total SCFAs. Butyrate, one of the main SCFAs, plays critical roles in host health, including energy metabolism, intestinal homeostasis, immune system regulation, and gut-brain axis modulation [31]. The major butyrate producers in the gut microbiota were reported to be depleted in young adults with depression [32]. Likewise, *Faecalibacterium prausnitzii*, a major butyrate producer member, persistently decreased in inflammatory bowel disease (IBD) and is recognized as a common microbial disease biomarker [33,34]. Moreover, preconditioning macrophages with butyrate stimulates their antimicrobial potency, limiting the expansion of gut bacteria and enhancing gut resistance to enteric invasion [35].

We recently reported that several classes of antidepressants possess selective antimicrobial activity against key commensal gut microbes [6]. The direct antimicrobial effect of psychotropic drugs has also emerged as a potential antidepressant mechanism of action through the modulation of the gut microbiota. For example, ketamine and lanicemine, NMDAR antagonists, exhibit distinct pharmacological effects in treating depression-resistant patients, with ketamine having fast and long-lasting antidepressant effects, whereas lanicemine is inactive [36]. The authors also found that ketamine altered the fecal microbiome composition in a mouse model of chronic social defeat stress [36], supporting the hypothesis that gut microbiota modulation could be a part of the ketamine antidepressant mode of action, and that the antimicrobial action of psychotropic compounds could mitigate gut dysbiosis in patients with MDD [37]. However, long-term antidepressant use with antimicrobial effects has been linked to several side effects, including microbial resistance and *C. difficile* infections [38]. Furthermore, our study showed that psychotropic drugs have the potential to induce dysbiosis, resulting in a deleterious shift in microbiome structure and metabolism. Long-term use of psychotropic chemicals with antimicrobial properties may lead to adaptive or differential specific impairments in the gut microbiome genera, which are critically linked to human health and disease. As patients are usually on chronic antidepressant therapy, the impact of psychotropic drugs should be considered a confounding factor in microbiome biomarker studies [6]. Therefore, there is an urgent need to better understand the associated antimicrobial mechanisms and the impact of psychotropic agents on the structure and metabolism of gut microbiota.

Although utilizing an ex vivo system to replicate the human gut may introduce confounding variables that make it challenging to draw biological conclusions, this system offers the advantage of testing different treatments on a highly controlled microbiome-based structure. Moreover, using complex fecal communities in ex vivo models can provide valuable insights into the short-term effects of xenobiotics and probiotics on the digestive system. However, it is important to note that using fecal samples from a single individual cannot account for inter-individual variations in microbiome composition that could impact stress recovery. Additionally, our incubation times only reflect a two-day exposure, which limits our understanding of the long-term effects of antidepressants and probiotics on stress recovery. Despite these limitations, our findings suggest that a specific probiotic mixture can positively affect gut microbiome recovery following antidepressant treatment in an ex vivo setting. To confirm these findings, future cohort studies should consider the personalized nature of the gut microbiota and examine the lack of immunological and other physiological modulatory factors in the ex vivo system.

In conclusion, psychotropics, notably aripiprazole, induced significant alterations in human gut microbiome composition and metabolic profile, eventually leading to gut dysbiosis. These observed changes were consistent with previous in vitro and in vivo data on the inhibitory effects of psychotropics, highlighting the validity of the ex vivo simulated human colon for studying their impact on gut microbiota. The probiotic mixture of *L. rhamnosus* HA-114 and *B. longum* R0175 exhibited a protective effect and alleviated gut dysbiosis induced by psychotropics, indicating that probiotics are beneficial in maintaining gut homeostasis. Our results shed light on the adverse effects of psychotropic medications and the potential of probiotics to mitigate associated gut dysbiosis.

## 4. Materials and Methods

### 4.1. Psychotropics and Probiotics

Two psychotropics were investigated in this study, aripiprazole (atypical antipsychotics) and (S)-citalopram oxalate (Serotonin-specific reuptake inhibitors antidepressant), both purchased from TCI America (Portland, OR, USA). These two drugs were selected following the demonstration of potentially antimicrobial effect within intestinal bacterial pure cultures from our previous work [6]. Stock solutions of each drug at a 20 mg/mL concentration were prepared according to the manufacturer’s recommendations, filter-sterilized (0.22 µm), and then stored at −20 °C until use. *Lacticaseibacillus* (*L*.) *rhamnosus* HA-114 and *Bifidobacterium* (*B*.) *longum* R0175 were kindly supplied by Lallemand Health Solutions (Montreal, QC, Canada) and used hereafter as a probiotic mixture (ratio 1:1). The strains were cultured from the lyophilized powder in MRS broth (BD Difco) supplemented with 0.1% HCL-cysteine and maintained at −20 °C until use. Before each experiment, the bacteria were sub-cultured three times anaerobically at 37 °C with no shaking for 24 h each to obtain a robustly and uniformly growing culture.

### 4.2. Feces Collection and Microbiota Immobilization

Fecal samples were collected fresh from two unrelated healthy adult donors (D1: female; age 36 and D2: male; age 39). The volunteers were not suffering from any known colonic conditions, were not taking pre- or probiotic supplements, and had not received any psychotropics (no psychiatric illness) or antibiotics treatment for at least three months prior to sample collection. The two donors provided their written, informed consent. The sampling of fecal material for inoculation of colonic fermentation has been approved by the University of Ottawa research ethics board (certificate H-02-18-347; 29 July 2019). The feces were processed immediately after arriving at the lab to slurries by dilution in reduced peptone water (20%, *w*/*v*), homogenized, and further immobilized in 1–2 mm gel beads consisting of gellan gum (2.5%, *w*/*v*), xanthan (0.25%, *w*/*v*), and sodium citrate (0.2%, *w*/*v*) under anaerobic conditions as described previously in details [10,39]. The immobilized microbial community from each donor was used to run one independent fermentation experiment.

### 4.3. Experimental Set-Up and Fermentation Procedure

**Figure 7:**
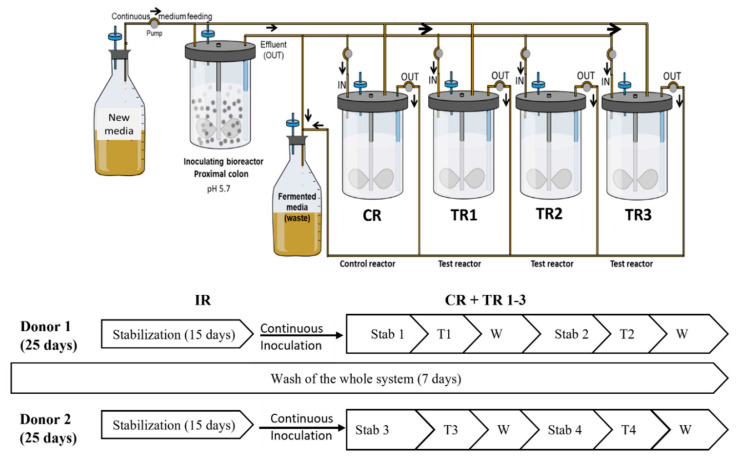
Experimental reactor set-up of the ex vivo fermentation model. IR: inoculum reactor, containing immobilized donor feces (30% *v*/*v*); CR: control reactor; TR1-TR3: test reactors 1–3; Stab: stabilization period (2 days); T: treatment period (2 days); W: wash period (1 day).

The continuous fermentation of two replicates of each donor microbiome was carried out for 25 days using an ex vivo model simulating the large intestine (N*u*GUT Research Platform, University of Ottawa) as previously described [10]. This model consists of a two-stage design comprising an inoculation reactor (IR) with immobilized fecal microbiota used to continuously inoculate four second-stage reactors operated in parallel (Figure 7). Each reactor was set up to reproduce the physiological and microbiological conditions of the adult proximal colon (pH 5.7, stirring at 120 rpm, 37 °C, and mean retention time of 8 h). Anaerobiosis was ensured through continuous headspace flushing of N_2_ and CO_2_ at a 0.9:0.1 ratio, and a constant pH of 5.7 was maintained by adding 2.5 M NaOH.

The fermentation procedure was initiated by transferring 60 mL of immobilized gel beads into the IR (1L BioFlo^®^ 120 vessel; Eppendorf, Mississauga, ON, Canada) containing 140 mL fresh sterile Macfarlane culture medium prepared as previously described [40]. During the first 48 h, the colonic model was run as a batch culture to allow beads colonization, and the nutritive medium was replaced by a fresh medium every 12 h. Following the 48 h, the medium flow was switched to continuous mode for the rest of the experiment. After a stabilization period of 15 days, the microbial community in IR was used to inoculate four-second stage DASGIP^®^ bioreactors (Eppendorf, Mississauga, ON): one CR (Control reactor: non-treatment control) and three treatment reactors TR1-3 that were challenged with different parallel treatment periods (Figure 7). The working volume of 100 mL was maintained in each sub-bioreactor by inoculation of 5% (*v*/*v*) (0.62 mL/h) IR effluent and the addition of 95% (*v*/*v*) (11.88 mL/h) fresh fermentation medium. Then, the entire second stage bioreactors were run for another 2 days to reach the stability of the microbial community. Once stabilization is reached in all reactors, the bioreactors were subjected to treatment every 24 h as follow:

- CR bioreactor: served as non-treatment control
- TR1 bioreactor: challenged with aripiprazole or (S)-citalopram at a final concentration of 400 µg/mL, simulating an estimated single daily dose of psychotropics as discussed before in our previous study [6].
- TR2 bioreactor: challenged with the probiotic mixture (*L. rhamnosus* and *B. longum*) added at a final concentration of 10^9^ CFU/mL each.
- TR3 bioreactor: challenged with aripiprazole or (S)-citalopram at 400 µg/mL and the probiotic mixture.

Effluent samples (2 mL) were taken from bioreactors at 0, 2, 4, 6, 8, 12, 24, and 48 h of treatment. The collected samples were separated (centrifugation at 14,000× *g*, 5 min, 4 °C) and pellet used for metagenomic DNA extraction while the supernatant was used for short-chain fatty acids (SCFA) analysis. After 48 h of treatment, the four subreactors were disconnected, washed, autoclaved, and set up for a second replicate of inoculation by the same donor microbiome developed in the IR. In other words, each fermentation experiment was conducted in duplicate for each fecal sample donor.

### 4.4. Microbial Community Analyses

#### 4.4.1. DNA Extraction

Genomic DNA was extracted from the pellet of fecal slurry and fermentation samples using a Fast DNA Spin Kit (MP Biomedicals; Solon, OH, USA) following the manufacturer’s instructions. The mechanical lysis was performed in 2 cycles of 40 s each at a speed of 6.0 m/s in a Bead Mill-24 Homogenizer (Fisher Scientific; Ottawa, ON, Canada) with 5 min cooling on ice between the two cycles [10]. The amount of extracted DNA was quantified using the Qubit fluorometer (Invitrogen; Carlsbad, CA, USA) and stored at −20 °C until used for further analysis.

#### 4.4.2. 16S rRNA Gene Sequencing

The microbial profile of fecal slurry and fermentation samples was assessed using tag-encoded multiplexed paired-end 16S rRNA gene MiSeq sequencing (Illumina, CA, USA). The V3-V4 regions of the 16S rRNA gene were amplified using dual-barcoded primers, and the amplicon library for sequencing was constructed using Illumina standard protocol. The amplicon libraries were pooled in equimolar amounts and paired-end sequenced with Illumina MiSeq platform (N*u*GUT Research Platform, University of Ottawa) using 600 bp MiSeq Reagent Kit v3 (Illumina; San Diego, CA, USA) as per standard protocol.

Sequences were quality filtered and denoised using the DADA2 pipeline [10] and clustered into observed features based on 97%-similarity using the Greengenes database (v13.8) via QIIME 2.2020.6 software [41]. The observed features were rarefied into an equal number of 12,000 reads per sample using QIIME 2.2020.6. Changes in alpha diversity were estimated with observed features, Shannon entropy, Pielou_Evenness, and Faith_pd. Beta-diversity among samples was calculated using the Bray-Curtis distance and visualized using principal coordinate analysis (PCoA). The contribution of different treatments to the diversity of the gut microbiota community was assessed from the Bray-Curtis distance matrix using the permutational multivariate analysis of variance (PERMANOVA)-pairwise and 999 permutations [41]. To identify differential taxa among different treatments, linear discriminant effect size analysis was conducted on the relative abundance of different taxa levels [42]. Samples were labeled with the treatment type as the sample class and the time points as the subclass. Taxa with log_10_ LDA score ≥ 2 and *p* < 0.05 were considered significant. Analysis of Compositions of Microbiome (ANCOM) was used to validate the LEfSe-identified significant differential taxa (Supplementary Table S6). When required Mann-Whitney test or Kruskal–Wallis test was applied for statistical analysis, and *p*-values were corrected using the two-stage Benjamini, Krieger, and Yekutieli false discovery rate (FDR) procedure.

#### 4.4.3. Detection and Quantification of Viable Probiotics with PMA-qPCR

The viability of probiotics was assessed after 0, 4, 8, 12, 24, and 48 h of treatment. To avoid DNA amplification from dead cells, PMA treatment was added to a 500 μL sample to a final concentration of 50 μM and thoroughly mixed on a vortex. The samples were incubated in the dark for 5 min (mixing to each 1 min). Then the samples were placed under the light of a lamp for 5 min, centrifuged (14,000 g, 5 min), and proceeded to DNA extraction as described above. Next, qPCR was performed using SsoAdvanced Universal SYBR Green Supermix (Bio-rad; Mississauga, ON, Canada), with analysis in Bio-Rad CFX Maestro software. Specific primers were selected to detect bacteria of interest and are shown in Supplementary Table S7. Each sample was tested in duplicate in a total volume of 20 µL per reaction. 25 ng of template DNA was added to a reaction mixture containing 0.25 µM of each primer and 1xSsoAdvanced Universal SYBR Green Supermix. Each sample was analyzed in duplicate. Amplification conditions 3 min at 98 °C followed by 40 cycles of 95 °C for 15 s and 60 °C for 1 min with data collection at the second step of each cycle. The standard curve was generated using a 10-fold serial dilution of genomic DNA or 16 S rRNA gene (10^8^−10^0^ copies per μL) from the respective microorganisms’ pure cultures. Non-template control (NTC) samples were carried out in all qPCR runs as a negative control. The mean values from assays of probiotics viability were expressed in log CFU equivalent/mL.

#### 4.4.4. Determination of SCFAs Content

The production of short-chain fatty acids (SCFAs; butyrate, acetate, and propionate) in fermentation samples from all sub-reactors was determined using Gas Chromatography coupled to Flame Ionization Detector (GC-FID) (Shimadzu GC-2030) as previously described [10]. In brief, supernatants collected from fermentation samples were centrifuged (14,000 g, 30 min, 4 °C) and filter sterilized (0.22 µm). 2-ethyl butyric acid was used as an internal standard and added to each sample at a concentration of 0.5 mM. Around 100 µL of each sample was injected in a capillary column Stabilwax-DA (60 m × 0.25 µm; Restek) with a run time of 20 min. The peaks were identified and quantified with standards from MilliporeSigma (Oakville, ON, Canada). Results were expressed as the concentration of SCFAs in mM. All samples were analyzed in duplicates (two technical measures).

### 4.5. Statistical Analyses

Data from Gas Chromatography analyses were analyzed using GraphPad Prism v8.3. (GraphPad Software, San Diego, CA, USA) in order to assess the significance of results among treatments at the same time and among different time points within each treatment. Data are expressed as means ± Standard deviations (SD), and an ANOVA test with Bonferroni as a post hoc test for multiple comparisons was used (*p*-values < 0.05).

## Data Availability

The dataset of the 16S rRNA sequences is available at: https://www.ncbi.nlm.nih.gov/bioproject/ under the accession number PRJNA792159 and PRJNA792161 (accessed on 14 April 2023).

## Supplementary Materials

The following supporting information can be downloaded at: https://www.mdpi.com/article/10.3390/ijms24087326/s1.
