# Supplementary material for: Evidence of the Dysbiotic Effect of Psychotropics on Gut Microbiota and Capacity of Probiotics to Alleviate Related Dysbiosis in a Model of the Human Colon
Source: Int J Mol Sci. 2023 Apr 15;24(8):7326. doi: 10.3390/ijms24087326 (PMC10138884; doi:10.3390/ijms24087326)
Supplement: Supplementary file 1 [file ijms-24-07326-s001.zip › Supplementary figures and Tables.pdf]

Supplementary files:

Evidence of the dysbiotic effect of psychotropics on gut microbiota and capacity of probiotics to alleviate related dysbiosis in a model of the human colon

Yasmina Ait Chait[1#], Walid Mottawea[1,2#], Thomas A. Tompkins[3], Riadh Hammami[1,4,*]

[1]N*u*Gut Research Platform, School of Nutrition Sciences, Faculty of Health Sciences, University of Ottawa, Ottawa, Ontario, K1N 6N5, Canada.

[2] Department of Microbiology and Immunology, Faculty of Pharmacy, Mansoura University, Mansoura, Egypt.

[3]Rosell Institute for Microbiome and Probiotics, Montreal, Quebec, H4P 2R2, Canada.

[4] Department of Biochemistry, Microbiology and Immunology, Faculty of Medicine, University of Ottawa, Ottawa, Ontario, Canada

# Those authors Contributed equally to this work.

*Corresponding author: riadh.hammami@uottawa.ca

A)

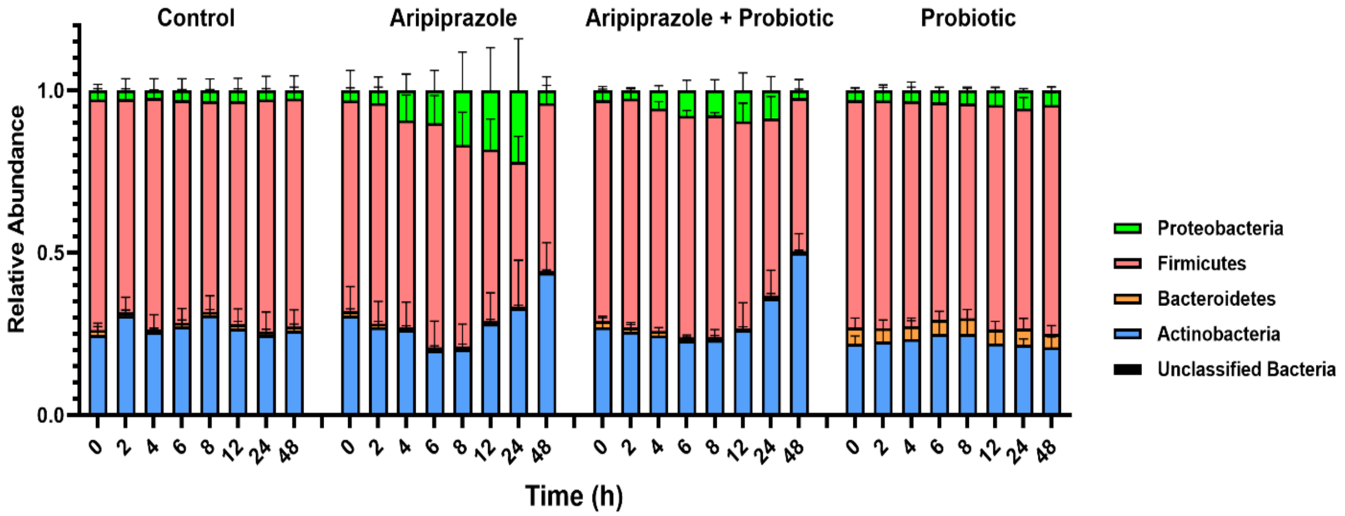

B)

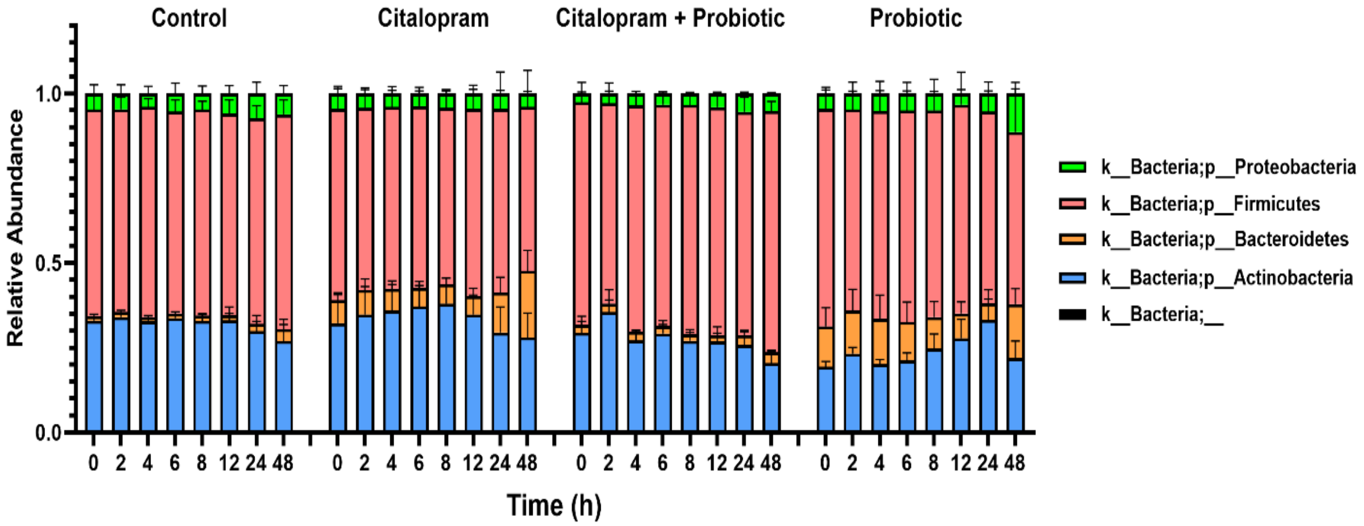

Figure S1: Microbial community composition. Bar plot of the relative abundances at the phylum level developed in different bioreactors of colonic fermentation model over 48 hours. **A:** effect of aripiprazole treatment; **B:** effect of (S)-citalopram treatment.

Table S1: Statistics of ADONIS permutation-based (999 permutations) multivariate analysis of variance for the effect of treatment and time on samples beta-diversity based on Bray-Curtis distances.

**Aripiprazole**		**F.Model**	**R2**	**Pr(>F)**
Donor 1	TREATMENT	9.307579	0.371074	0.001
	TIME	6.305293	0.062845	0.001
	TREATMENT:TIME	2.198911	0.087666	0.018
	Residuals	NA	0.478415	NA
	Total	NA	1	NA
Donor 2	TREATMENT	5.927083	0.281889	0.001
	TIME	2.93144	0.034854	0.022
	TREATMENT:TIME	2.866356	0.136322	0.002
	Residuals	NA	0.546934	NA
	Total	NA	1	NA
**Citalopram**				
Donor 1	TREATMENT	4.195936	0.378395	0.001
	TIME	0.704147	0.101601	0.895
	TREATMENT:TIME	0.340482	0.141243	1
	Residuals	NA	0.378761	NA
	Total	NA	1	NA
Donor 2	TREATMENT	4.987755	0.281376	0.001
	TIME	1.080921	0.106712	0.382
	TREATMENT:TIME	0.965978	0.245224	0.53
	Residuals	NA	0.366687	NA
	Total	NA	1	NA

Table S2: Log CFU/ml of *L. rhamnosus* HA-114 added alone in all treatments (reactors with probiotics only)

Time (h)	Aripiprazole		(S)-citalopram	
	Donor 1	Donor 2	Donor 1	Donor 2
**0**	9.29	8.97	8.83	8.91
**4**	9.09	9.00	8.86	8.89
**8**	9.08	8.15	8.88	8.87
**12**	8.92	8.27	8.75	8.81
**24**	8.91	7.91	8.81	8.70
**48**	8.87	8.47	8.67	8.72

Table S3: Log CFU/ml of *L. rhamnosus* HA-114 added with aripiprazole / (S)-citalopram treatments

Time (h)	Aripiprazole		(S)-citalopram	
	Donor 1	Donor 2	Donor 1	Donor 2
**0**	9.21	8.92	8.82	8.93
**4**	9.20	8.75	8.70	8.91
**8**	9.08	8.54	8.66	8.85
**12**	9.14	8.63	8.70	8.80
**24**	8.98	8.76	8.44	8.69
**48**	8.98	8.68	8.46	8.69

Table S4: Log CFU/ml of *B. longum* R0175 added alone in all treatments (reactors with probiotics only)

**Time (h)**	**Aripiprazole**		**(S)-citalopram**	
	**Donor 1**	**Donor 2**	**Donor 1**	**Donor 2**
**0**	9.16	8.91	8.80	8.85
**4**	8.92	9.02	8.86	8.88
**8**	8.85	8.55	8.84	8.87
**12**	9.05	8.38	8.75	8.84
**24**	8.68	8.43	8.73	8.77
**48**	9.09	8.39	8.60	8.76

Table S5: Log CFU/ml of *B. longum* R0175 added alone with aripiprazole / (S)-citalopram treatments

**Time (h)**	**Aripiprazole**		**(S)-citalopram**	
	**Donor 1**	**Donor 2**	**Donor 1**	**Donor 2**
**0**	9.12	8.84	8.51	8.85
**4**	9.11	9.38	8.76	8.87
**8**	8.87	8.75	8.77	8.83
**12**	8.81	8.64	8.75	8.79
**24**	8.57	8.26	8.66	8.68
**48**	8.67	8.66	8.70	8.61

Table S6: Differentially abundant taxa between different treatments of Aripiprazole and Citalopram identified by Analysis of Compositions of Microbiome (ANCOM) statistics.

**Aripiprazole**	**W**
k__Bacteria;p__Firmicutes;c__Bacilli;o__Lactobacillales;f__Lactobacillaceae	47
k__Bacteria;p__Firmicutes;c__Clostridia;o__Clostridiales;f__Veillonellaceae	41
k__Bacteria;p__Firmicutes;c__Clostridia;o__Clostridiales;f__Lachnospiraceae	39
k__Bacteria;p__Firmicutes;c__Bacilli;o__Lactobacillales;f__Lactobacillaceae;__	91
k__Bacteria;p__Firmicutes;c__Bacilli;o__Lactobacillales;f__Lactobacillaceae;g__Pediococcus	87
k__Bacteria;p__Firmicutes;c__Clostridia;o__Clostridiales;f__Veillonellaceae;__	82
k__Bacteria;p__Firmicutes;c__Bacilli;o__Lactobacillales;f__Lactobacillaceae;__;__	121
k__Bacteria;p__Firmicutes;c__Bacilli;o__Lactobacillales;f__Lactobacillaceae;g__Pediococcus;__	114
k__Bacteria;p__Firmicutes;c__Clostridia;o__Clostridiales;f__Veillonellaceae;__;__	110
**Citalopram**	
k__Bacteria;p__Firmicutes;c__Bacilli;o__Lactobacillales;f__Lactobacillaceae	58
k__Bacteria;p__Firmicutes;c__Bacilli;o__Lactobacillales;f__Lactobacillaceae;__	113
k__Bacteria;p__Firmicutes;c__Bacilli;o__Lactobacillales;f__Lactobacillaceae;g__Pediococcus	113
k__Bacteria;p__Actinobacteria;c__Coriobacteriia;o__Coriobacteriales;f__Coriobacteriaceae;g__Eggerthella	111
k__Bacteria;p__Firmicutes;c__Clostridia;o__Clostridiales;f__Ruminococcaceae;g__Subdoligranulum	108
k__Bacteria;p__Firmicutes;c__Clostridia;o__Clostridiales;f__Veillonellaceae;g__Anaerosinus	107
k__Bacteria;p__Firmicutes;c__Clostridia;o__Clostridiales;f__Lachnospiraceae;__	104
k__Bacteria;p__Firmicutes;c__Bacilli;o__Lactobacillales;f__Lactobacillaceae;__;__	156
k__Bacteria;p__Firmicutes;c__Bacilli;o__Lactobacillales;f__Lactobacillaceae;g__Pediococcus;__	156
k__Bacteria;p__Firmicutes;c__Bacilli;o__Lactobacillales;f__Lactobacillaceae;g__Pediococcus;s__damnosus	155
k__Bacteria;p__Firmicutes;c__Bacilli;o__Lactobacillales;f__Lactobacillaceae;g__Lactobacillus;s__ruminis	154
k__Bacteria;p__Actinobacteria;c__Coriobacteriia;o__Coriobacteriales;f__Coriobacteriaceae;g__Eggerthella;s__	152
k__Bacteria;p__Firmicutes;c__Clostridia;o__Clostridiales;f__Veillonellaceae;g__Anaerosinus;s__glycerini	148
k__Bacteria;p__Firmicutes;c__Clostridia;o__Clostridiales;f__Ruminococcaceae;g__Subdoligranulum;s__variabile	147
k__Bacteria;p__Firmicutes;c__Clostridia;o__Clostridiales;f__Lachnospiraceae;__;__	146

Table S7. Primer information *B. longum* R0175 and *L. rhamnosus* HA-114

Target strain	Primer name	Sequence (5’-3’)	Annealing temperature (°C)	Amplicon size (bp)
B. longum **R0175**	R175_AP_HP10_F	GTCGCCACATTTCATCGCAA	60	99
	R175_AP_HP10_R	GAGAGCTTCGATTGGCGAAC		
L. rhamnosus **HA-114**	113A29_293FL	ACTCCAAAGAGCATTACCTCCG	60	71
	113A29_321RU	TGAATATGCCGGATCTAAGTCCA		
